# Three Sudden Cardiac Deaths Associated with Lyme Carditis — United States, November 2012–July 2013

**Published:** 2013-12-13

**Authors:** Gregory Ray, Thadeus Schulz, Wayne Daniels, Elizabeth R. Daly, Thomas A. Andrew, Catherine M. Brown, Peter Cummings, Randall Nelson, Matthew L. Cartter, P. Bryon Backenson, Jennifer L. White, Philip M. Kurpiel, Russell Rockwell, Andrew S. Rotans, Christen Hertzog, Linda S. Squires, Jeanne V. Linden, Margaret Prial, Jennifer House, Pam Pontones, Brigid Batten, Dianna Blau, Marlene DeLeon-Carnes, Atis Muehlenbachs, Jana Ritter, Jeanine Sanders, Sherif R. Zaki, Paul Mead, Alison Hinckley, Christina Nelson, Anna Perea, Martin Schriefer, Claudia Molins, Joseph D. Forrester

**Affiliations:** Cryolife, Inc. Kennesaw, GA; New Hampshire Dept of Health and Human Svcs; New Hampshire Office of the Chief Medical Examiner; Massachusetts Dept of Public Health; Massachusetts Office of the Chief Medical Examiner; Connecticut Dept of Public Health; New York State Dept of Health; Dutchess County Dept of Health; Wadsworth Center, New York State Dept of Health; Office of the Medical Examiner, Orange County, New York; Indiana State Dept of Health; Div of High-Consequence Pathogens and Pathology; Div of Vector-Borne Infectious Diseases, National Center for Emerging and Zoonotic Infectious Disease; EIS Officer, CDC

Lyme disease* is a multisystem illness caused by *Borrelia burgdorferi*, a spirochete transmitted by certain species of *Ixodes* ticks. Approximately 30,000 confirmed and probable cases of Lyme disease were reported in the United States in 2012, primarily from high-incidence states in the Northeast (Connecticut, Delaware, Maine, Maryland, Massachusetts, New Hampshire, New Jersey, New York, Pennsylvania, Rhode Island, and Vermont) and upper Midwest (Minnesota and Wisconsin) (1,2).† Common manifestations include cutaneous, neurologic, and rheumatologic signs and symptoms. Symptomatic infection of the heart is rare in recognized Lyme disease cases and usually resolves promptly with appropriate antibiotic therapy. Nonetheless, cardiac involvement occasionally can cause life-threatening cardiac conduction abnormalities. During November 2012–July 2013, one woman and two men (ranging in age from 26 to 38 years) from high-incidence Lyme disease states experienced sudden cardiac death and, on postmortem examination, were found to have evidence of Lyme carditis. The three deaths were investigated by the Connecticut Department of Public Health, Massachusetts Department of Public Health, New Hampshire Department of Public Health, New York State Department of Health, and CDC. Donated corneas from two decedents had been transplanted to three recipients before the diagnosis of Lyme disease was established, but no evidence of disease transmission was found. Although death from Lyme carditis is rare, it should be considered in cases of sudden cardiac death in patients from high-incidence Lyme disease regions. Reducing exposure to ticks is the best method for preventing Lyme disease and other tickborne infections.§

## Case Reports and Public Health Investigation

### Patient 1

In November 2012, a Massachusetts resident was found unresponsive in an automobile after it veered off the road. No evidence of traumatic injury was found. An electrocardiogram (EKG) performed by emergency responders showed no cardiac activity, and the patient was pronounced dead at a nearby hospital. The patient had no serious preexisting medical conditions. No rash was noted at autopsy, although some atherosclerosis was present. Interviews with next-of-kin revealed that the patient had described a nonspecific illness with malaise and muscle and joint pain during the 2 weeks preceding death. The patient lived alone with a dog that was reported to have ticks frequently.

The decedent’s corneas and skin, musculoskeletal, cardiac, and vascular tissues were recovered for potential transplantation. The heart was sent to tissue bank A for valve recovery. Microscopic examination of cardiac tissue found extensive myocarditis with mixed perivascular lymphoplasmacytic inflammation suggestive of Lyme carditis. A postmortem serum sample tested at CDC yielded serologic evidence of recent infection with *B. burgdorferi*, reacting strongly in both whole cell sonicate (WCS) and C6 enzyme immunoassay (EIA), and against all three scored bands (23 kDa, 39 kDa, and 41 kDa) by immunoglobulin M (IgM) Western blot. Western blot testing for immunoglobulin G (IgG) antibodies demonstrated reactivity against four of 10 scored bands (23 kDa, 39 kDa, 41 kDa, and 45 kDa); these serologic findings were consistent with early disseminated Lyme disease.

**FIGURE 1:**
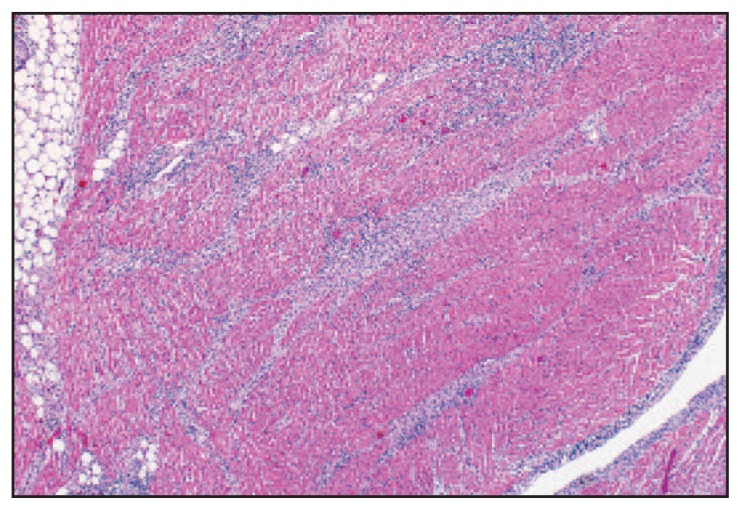
Hematoxylin and eosin stain at 6.25× magnification demonstrating interstitial perivascular lymphoplasmacytic pancarditis in postmortem tissue of one of three patients whose death was associated with Lyme carditis — United States, 2013

**FIGURE 2:**
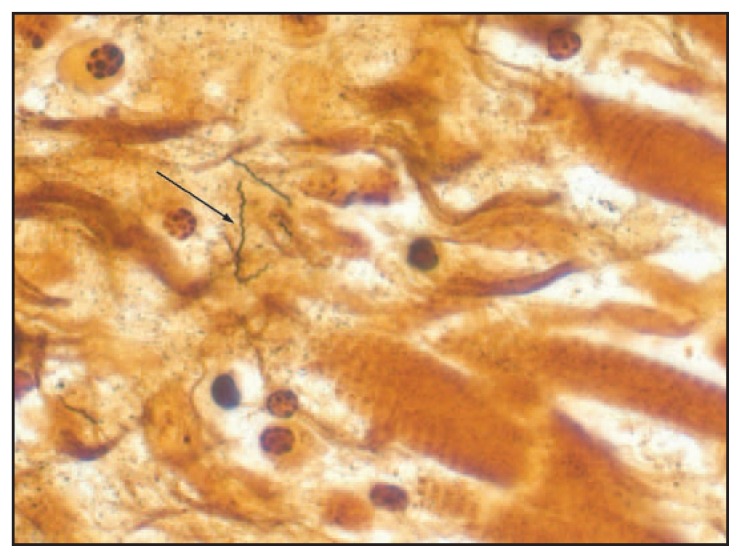
Warthin-Starry stain of cardiac tissue at 158× magnification demonstrating *Borrelia burgdorferi* spirochetes (arrow) in one of three patients whose death was associated with Lyme carditis — United States, 2013

Histopathologic evaluation of postmortem tissues at CDC also was suggestive of Lyme pancarditis (Figure 1) and abundant spirochetes were observed by Warthin-Starry silver stain (Figure 2). Spirochetes also were detected in the myocardium by immunohistochemistry (IHC). Polymerase chain reaction (PCR) assays detected *B. burgdorferi* in extracts of formalin-fixed, paraffin-embedded heart tissue based on outer surface protein A, flagellin, and plasminogen-binding protein gene targets. No donor tissues were transplanted.

### Patient 2

In July 2013, a New York state resident experienced chest pain and collapsed at home. Cardiopulmonary resuscitation was unsuccessful, and the patient was pronounced dead at a local hospital. The patient’s past medical history included a diagnosis of Wolff-Parkinson-White syndrome, a cardiac conduction abnormality. The patient had no known tick contact or rash but was reported to be a hiker. Evidence of hypertensive and atherosclerotic cardiovascular disease was noted at autopsy. The decedent’s corneas and skin, musculoskeletal, vascular, and cardiac tissue were recovered for potential transplantation. Examination of cardiac tissue at tissue bank A revealed moderate diffuse, perivascular lymphoplasmacytic pancarditis, similar to that seen in patient 1. Serologic testing at CDC was consistent with recent infection with *B. burgdorferi*; WCS and C6 EIAs were strongly reactive, IgM Western blot demonstrated strong reactivity to all three scored bands, and IgG Western blot demonstrated reactivity to four scored bands (23 kDa, 41 kDa, 58 kDa, and 66 kDa). Rare spirochetes were identified in cardiac tissue by Warthin-Starry silver stain and IHC; heart tissues tested positive for *B. burgdorferi* by PCR.

Before diagnosis of *B. burgdorferi* infection, the decedent’s corneas were transplanted to two recipients. The transplanting physicians and cornea recipients subsequently were notified of the donor’s infection. Neither recipient 1 nor recipient 2 reported signs or symptoms of Lyme disease or problems with the transplanted cornea. Both recipients elected to receive antibiotic therapy with doxycycline. None of the remaining donated tissues were transplanted.

### Patient 3

In July 2013, a Connecticut resident collapsed while visiting New Hampshire and was pronounced dead at a local hospital. The patient had complained of episodic shortness of breath and anxiety during the 7–10 days before death. No rash, arthralgia, or neurologic symptoms were noted. A physician consulted 1 day before death prescribed clonazepam for anxiety; an EKG was not performed, nor were any antibiotics prescribed. The patient lived on a heavily wooded lot and had frequent tick exposure; there was no known history of cardiovascular disease. Autopsy revealed myocarditis, and the medical examiner submitted heart tissues to CDC for evaluation of suspected viral myocarditis. Corneas and skin were recovered for donation, and one cornea was transplanted to recipient 3. No other tissue was transplanted. Recipient 3 was examined 1 week after corneal transplant and was recovering as anticipated. Examination of heart tissues at CDC again demonstrated diffuse mixed perivascular lymphoplasmacytic pancarditis. Warthin-Starry stain revealed spirochetes in the myocardium, and IHC and PCR assays confirmed the spirochete as *B. burgdorferi.* WCS and C6 EIAs were positive, IgM Western blot was positive for all three scored bands, and IgG Western blot demonstrated reactivity to one scored band (41 kDa).

The eye bank was informed of the Lyme disease status of the donor and the recommendations for therapy. Before notification of the Lyme disease status of the donor, recipient 3 died of unrelated causes. No tissues or serum from recipient 3 were available for evaluation.

### Editorial Note

This report describes three cases of sudden cardiac death associated with Lyme carditis, with subsequent transplantation of corneas from two of the decedents into three recipients. Only rarely has death been attributed to Lyme carditis (3–6), and review of pathology reports at tissue bank A did not identify any additional confirmed cases among 20,000 cardiac specimens received since 2004. Whether the preexisting heart conditions found in two patients increased their risk for death is unclear.

What is already known on this topic?

Carditis with heart block is a known but uncommon complication of early disseminated Lyme disease that is generally treated effectively with appropriate antibiotic therapy. Four deaths from Lyme carditis have been reported.

What is added by this report?

This report describes three new cases of sudden cardiac death associated with Lyme carditis. The decedents were aged 26 to 38 years and lived in high-incidence Lyme disease areas.

What are the implications for public health practice?

Pathologists and medical examiners should be aware that Lyme carditis can be a cause of sudden cardiac death. All suspected cases of fatal Lyme carditis should be reported to state or local public health authorities, and the cases should be investigated. Physicians and health-care providers should ask patients with suspected Lyme disease about cardiac symptoms, and conversely, ask patients with acute, unexplained cardiac symptoms about possible tick exposure and symptoms of Lyme disease. Clinicians should encourage all patients to practice recommended tick bite prevention strategies.

*Borrelia burgdorferi* has been shown to affect all layers of the heart, but tends to spare the great vessels and heart valves (7). Inflammation is characteristically diffuse, perivascular, lymphohistiocytic, and plasma cell-rich. Spirochetes can be found within the myocardial cellular infiltrates; IHC and PCR testing can provide additional evidence of infection. Although Lyme carditis usually is present in conjunction with other features of the disease, such as erythema migrans, arthritis, or neurologic disease, it can be observed independently (8). The most common cardiac manifestation is atrioventricular block, which can fluctuate between first, second, and third degree (7,8). Second-degree or third-degree atrioventricular block occurs in approximately 0.8% of all Lyme disease cases reported to CDC (2). Symptoms of atrioventricular block, including lightheadedness, palpitations, shortness of breath, chest pain, and syncope can occur 4 days to 7 months after onset of disease, with a median of 21 days (7,8). With appropriate therapy (9), prognosis is excellent, and signs of cardiac involvement typically resolve within 1–6 weeks, depending on the degree of conduction disturbance (10). Some cases of complete heart block might require temporary pacing.

Although no cases of Lyme disease transmission through organ or tissue transplantation have been reported, the identification of organisms in tissue suggests the risk for transmission could exist. Ophthalmologic manifestations of Lyme disease are rare but can involve any of the ocular structures and occur during any stage of Lyme disease.¶ Given the rarity of ocular Lyme disease, and of corneal Lyme disease in particular, and the absence of ocular symptoms in the deceased patients, the need for antibiotics in this setting was equivocal. However, if administered, oral doxycycline would be expected to penetrate eye structures well.

Medical examiners and pathologists should be aware that Lyme carditis is a potential, albeit rare, cause for sudden cardiac death in persons from high-incidence Lyme disease areas. Diffuse, mixed perivascular lymphoplasmacytic infiltrates seen on pathologic examination of heart tissue from patients who have sudden cardiac death in high-incidence Lyme disease areas should prompt serologic evaluation for Lyme disease and further histopathologic examination for spirochetes, including IHC evaluation and PCR. Lyme disease is a nationally notifiable disease; all suspected cases of fatal Lyme carditis should be reported to state or local public health authorities, and the cases should be investigated.

Prompt recognition and early, appropriate therapy for Lyme disease is essential. Health-care providers should ask patients with suspected Lyme disease about cardiac symptoms and obtain an EKG if indicated. Conversely, they should ask patients with unexplained heart block about possible exposure to infected ticks. Health-care providers also should remind their patients of steps to prevent infection, including use of repellent, daily tick checks, prompt showering after potential exposure, and landscape management. The three deaths described in this report underscore the need for better methods of primary prevention of Lyme disease and other tickborne infections.

## Footnotes

* Additional information available at http://www.cdc.gov/lyme.

† Additional information regarding how many persons receive a diagnosis each year of Lyme disease is available at http://www.cdc.gov/lyme/faq/index.html#humancases.

§ Additional information available at http://www.cdc.gov/lyme/prev/index.html.

¶ Additional information available at http://www.sciencedirect.com/science/article/pii/S0161642099001281.
